# Supplementary figures and images for: Real-time analysis of nanopore-based metagenomic sequencing from infected orthopaedic devices
Source: BMC Genomics. 2018 Sep 27;19:714. doi: 10.1186/s12864-018-5094-y (PMC6161345; doi:10.1186/s12864-018-5094-y)

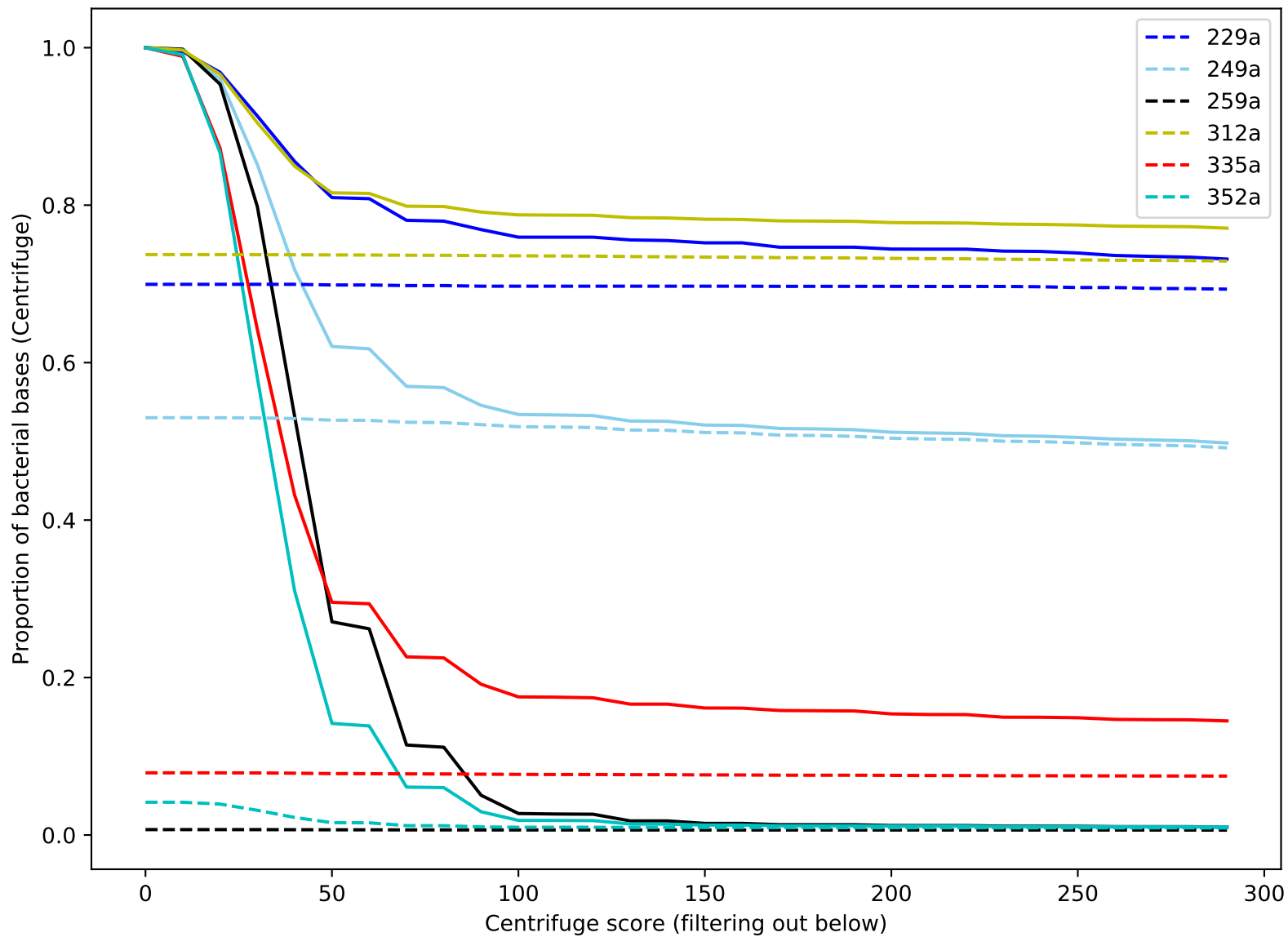

Supplement: Supplementary file 1 — Figure S1. Bases classified total or target over centrifuge score. Each sample has two lines of the same colour. The top line is total bacterial bases identified by centrifuge over the score threshold used. The second lower line is the validated detected species/infection for the sample (Target). As the score threshold increases, the number of total classified bases reduces at a great rate than the target bases, until a plateau and diminishing returns at approximately 150. (PDF 15 kb) [file 12864_2018_5094_MOESM1_ESM.pdf]

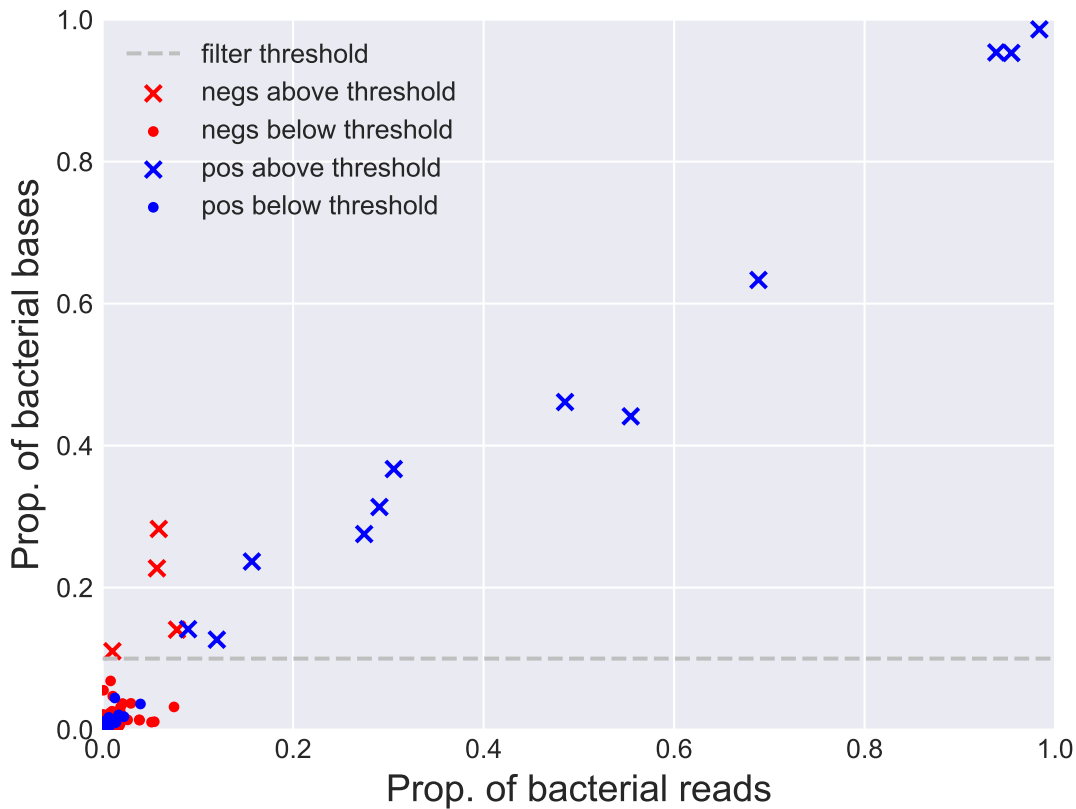

Supplement: Supplementary file 2 — Figure S2. Each species identified by centrifuge showing total bases over number of reads as proportions of total bacterial bases and total bacterial reads respectively. Species detections below the 0.1 proportion (i.e. less than 10%) of bases threshold are dots and species detections above the 0.1 proportion threshold are crosses. Culture negative controls are red and Culture negative positive samples are blue. (PDF 25 kb) [file 12864_2018_5094_MOESM2_ESM.pdf]

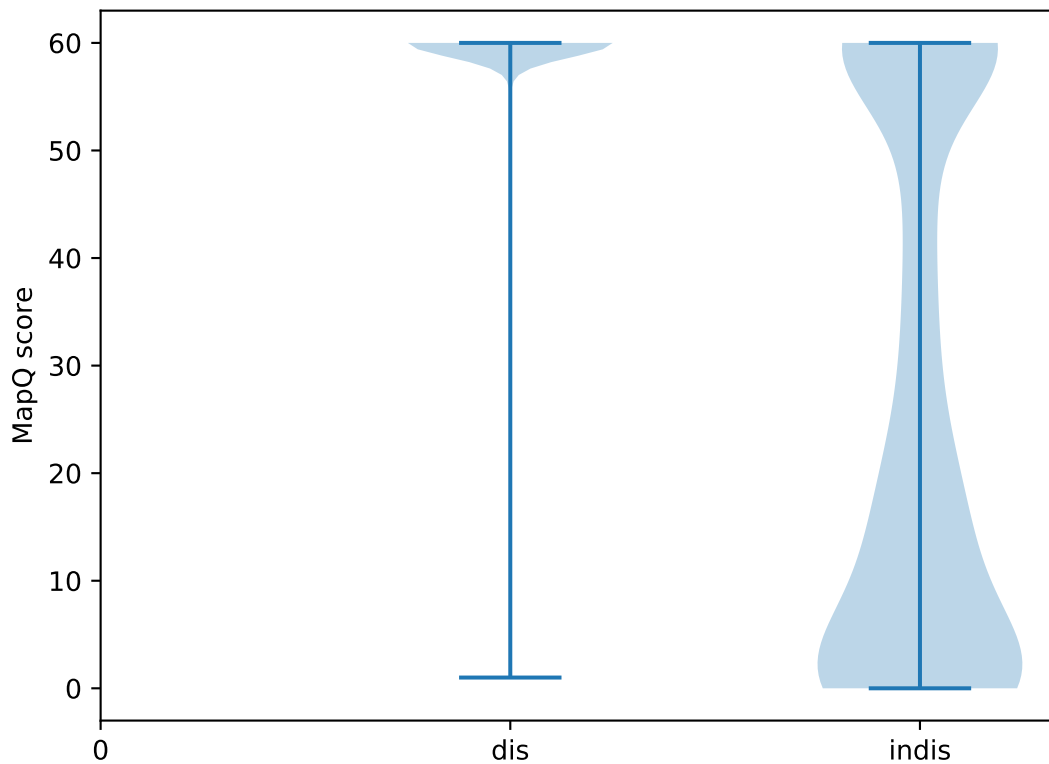

Supplement: Supplementary file 3 — Figure S3. Indiscriminate(indis) read and discriminate(dis) mapping qualities. Quality scores taken from mapping all reads to a reference with minimap2. Discriminate scores are from reads that have passed through the pipeline filtering thresholds and are determined to be reads specific to the reference. The indiscriminate are other reads that were likely to be host and/or contamination. (PDF 12 kb) [file 12864_2018_5094_MOESM3_ESM.pdf]

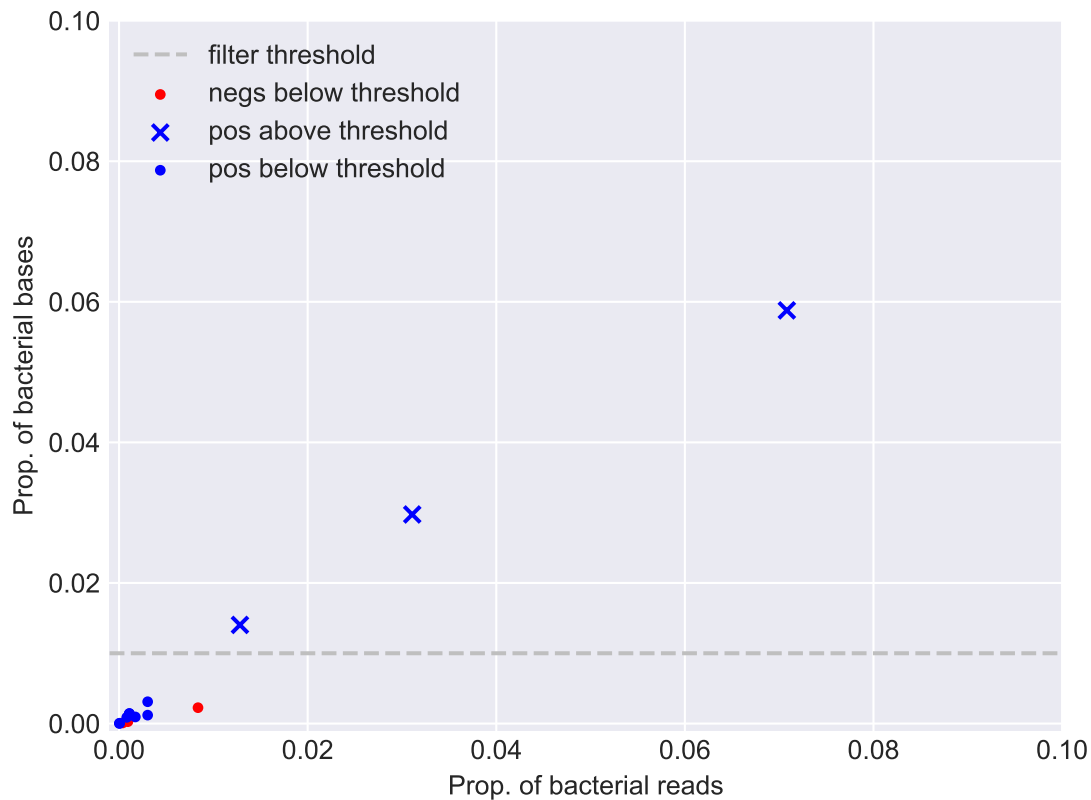

Supplement: Supplementary file 4 — Figure S4. Each species identified by minimap2 mapping showing total bases over number of reads as proportions of total bacterial bases (centrifuge) and total bacterial reads (centrifuge) respectively. Species detections below the 0.1 proportion (i.e. less than 1%) of bases threshold are dots and species detections above the 0.01 proportion threshold are crosses. Culture negative controls are red and Culture negative positive samples are blue. Shows shortened axis of below threshold hits. (PDF 13 kb) [file 12864_2018_5094_MOESM4_ESM.pdf]

# Task execution real-time

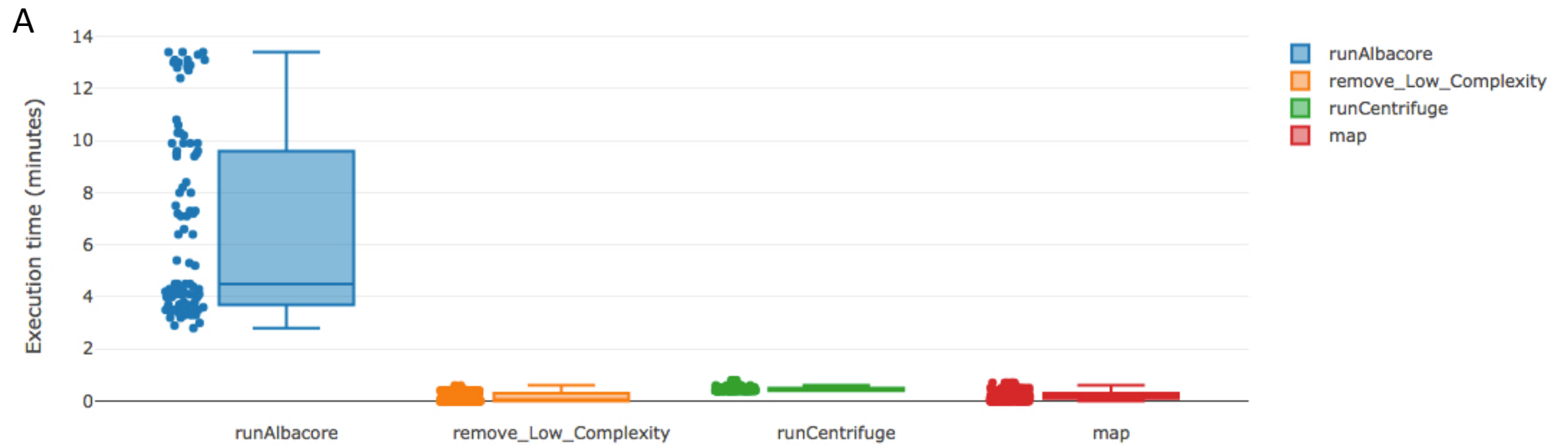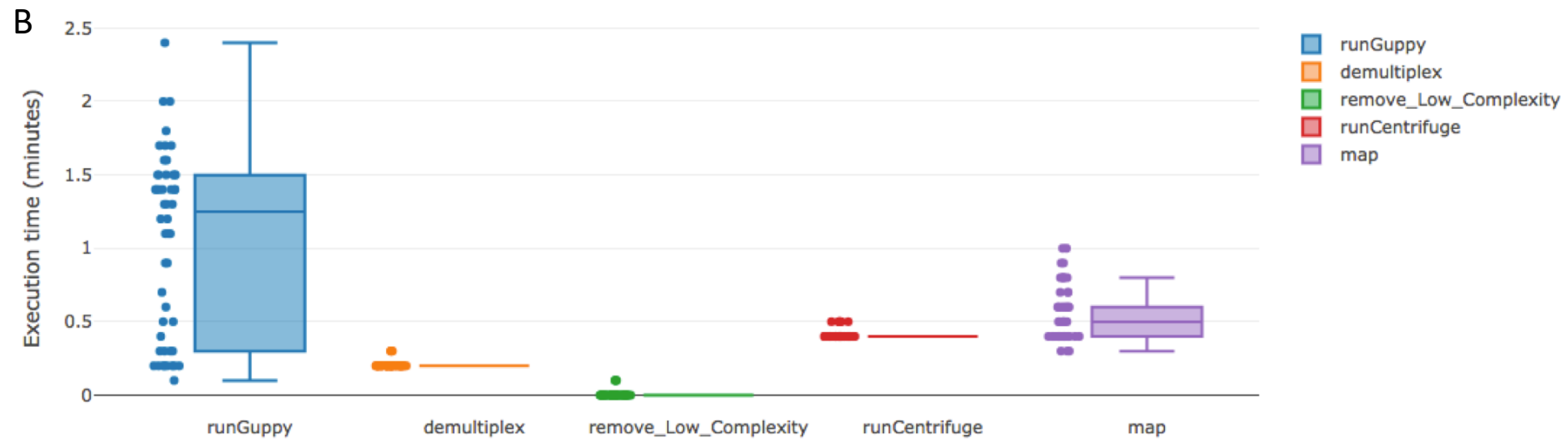

Task name

Supplement: Supplementary file 5 — Figure S5. Batch job duration times in minutes sample report taken from Nextflow. Using sample 354a as a representative for the bioinformatic analysis. (A) Batches were run over a heterogeneous SLURM cluster with variable node CPU speeds affecting Albacore performance. (B) Batches were run on a single machine with an Nvidia GTX 1050ti graphics card using guppy v0.3.0 for basecalling. (PDF 280 kb) [file 12864_2018_5094_MOESM5_ESM.pdf]
